# Increased dementia risk predominantly in diabetes mellitus rather than in hypertension or hyperlipidemia: a population-based cohort study

**DOI:** 10.1186/s13195-017-0236-z

**Published:** 2017-02-06

**Authors:** Yen-Chun Fan, Jung-Lung Hsu, Hong-Yi Tung, Chia-Chi Chou, Chyi-Huey Bai

**Affiliations:** 1 School of Public Health, College of Public Health, Taipei Medical University, Taipei, Taiwan; 2 Department of Neurology, Chang Gung Memorial Hospital Linkou Medical Center and College of Medicine, Chang-Gung University, Taoyuan, Taiwan; 3 Graduate Institute of Humanities in Medicine, Taipei Medical University, Taipei, Taiwan; 4 Brain and Consciousness Research Center, Taipei Medical University, Taipei, Taiwan; 5 Dementia Center and Section of Dementia, Department of Neurology, Chang Gung Memorial Hospital, Taoyuan, Taiwan; 6 Department of Gastroenterologic Surgery, Yuan’s General Hospital, Kaohsiung, Taiwan; 7 Department of Internal Medicine, Chang Gung Memorial Hospital, Keelung, Taiwan; 8 Department of Public Health, College of Medicine, Taipei Medical University, Taipei, Taiwan

**Keywords:** Diabetes mellitus, Hypertension, Hyperlipidemia, Dementia, National Health Insurance Research Database

## Abstract

**Background:**

The pathophysiology of insulin resistance-induced hypertension and hyperlipidemia might entail differences in dementia risk in cases with hypertension and hyperlipidemia without prior diabetes mellitus (DM). This study investigated whether incident hypertension, incident hyperlipidemia, or both, increased the dementia risk in patients with and without DM.

**Methods:**

A nationwide retrospective cohort study was conducted. The study sample was obtained from the National Health Insurance Research Database. We enrolled 10,316 patients with a new diagnosis of DM between 2000 and 2002 in the DM cohort. For the same period, we randomly selected 41,264 patients without DM in the non-DM cohort (matched by age and sex at a 1:4 ratio with the DM cohort). Both cohorts were then separately divided into four groups on the basis of incident hypertension or incident hyperlipidemia status.

**Results:**

In total, 51,580 patients aged between 20 and 99 years were enrolled. The dementia risk was higher in the DM cohort than in the non-DM cohort (adjusted hazard ratio (HR) = 1.47, 95% confidence interval (CI) = 1.30–1.67, *p* < 0.001). In the DM cohort, the dementia risk in patients with both hypertension and hyperlipidemia did not significantly increase compared with that in those without hypertension and hyperlipidemia (*p* = 0.529). Similar results were observed in those with either hypertension (*p* = 0.341) or hyperlipidemia (*p* = 0.189). In the non-DM cohort, patients with both hypertension and hyperlipidemia had a higher dementia risk (adjusted HR = 1.33, 95% CI = 1.09–1.63, *p* = 0.006). The results remained largely unchanged in patients with only hypertension (adjusted HR = 1.22, 95% CI = 1.05–1.40, *p* = 0.008). However, the dementia risk did not increase significantly in patients with only hyperlipidemia (*p* = 0.187).

**Conclusions:**

The development of hypertension, hyperlipidemia, or both, following a diagnosis of incident diabetes is secondary to diabetes onset and likely mediated through insulin resistance associated with diabetes, which does not further accentuate dementia risk. DM itself (i.e., the systemic influence of hyperglycemia) might be the main driver of increased dementia risk.

## Background

The exact pathogenesis underlying dementia remains elusive. Cardiovascular risk factors including diabetes mellitus (DM), hypertension, and hyperlipidemia are believed to be associated with dementia risk [1, 2]. In addition, the contribution of insulin resistance to these cardiovascular risk factors has been identified [3, 4]. The relationship between DM and dementia risk might be explained by the potential mechanism of insulin resistance [5, 6] that might be influenced by hyperglycemia [7]. Furthermore, patients with DM often present concomitant hypertension and dyslipidemia [8, 9].

Previous studies have reported an association of DM [10–13] and hypertension [14–16] with an elevated dementia risk. However, the association between hyperlipidemia and dementia remains debatable [17, 18]. These studies have been unable to determine whether the dementia risk is attributable to factors such as DM, hypertension, and hyperlipidemia. In addition, the development of DM, hypertension, and hyperlipidemia might share a common mechanism of insulin resistance [19], which initially presents as hyperglycemia [7]. Moreover, previous studies have not investigated the dementia risk associated with the sequence of occurrence of cardiovascular risk factors, especially of insulin resistance that is expressed DM [18, 20, 21].

The pathophysiology of insulin resistance-induced hypertension and hyperlipidemia might entail differences in dementia risk in cases with hypertension and hyperlipidemia without prior DM. In patients with diabetes, previously reported data indicate that insulin resistance might lead to the development of hypertension [22], which also contributes to elevated plasma triglyceride levels [23]. However, in patients without DM, hypertension and hyperlipidemia could be attributed to excess sodium consumption [24] and nutritional factors [25], respectively.

Therefore, we conducted a nationwide population-based cohort study up to a 10-year follow-up period to investigate whether patients with incident DM have a higher dementia risk. Furthermore, the possible dementia risk effect in development of hypertension, hyperlipidemia, or both, was examined in patients with incident diabetes.

## Methods

### Study design and data source

The data used in this retrospective cohort study were obtained from the Longitudinal Health Insurance Database 2005 (LHID2005), a subset of the National Health Insurance Research Database (NHIRD). The NHIRD, maintained by the National Health Research Institutes (NHRI), contains the claims data of beneficiaries enrolled in the Taiwan National Health Insurance (NHI) program, which was implemented in 1995 and covers approximately 99% of the 23 million residents of Taiwan. The NHRI compiles the claims data in the NHIRD for research purposes. All original claims data are anonymized and encrypted before being released to researchers. In addition, researchers who wish to use the NHIRD and its data subsets must sign a written agreement declaring that they have no intention to obtain information that could potentially violate patient privacy.

The LHID2005 contains the claims data of 1,000,000 beneficiaries randomly selected from the Registry of Beneficiaries of the NHIRD in 2005. The claims data include information related to outpatient visits, hospital admissions, and detailed prescriptions. Furthermore, according to the NHRI, the distribution of age, sex, and average insurance amount do not differ significantly between the LHID2005 and NHIRD.

The disease diagnoses in this study were defined according to the International Classification of Diseases, Ninth Revision, Clinical Modification (ICD-9-CM) codes. The diagnostic accuracy of diseases has been validated in the NHIRD [26, 27]. The prescribed drugs were identified according to the definition from the Anatomical Therapeutic Chemical (ATC) codes. This study was evaluated and approved by the Institutional Review Board of Taipei Medical University and Shin Kong Wu Ho-Su Memorial Hospital.

### Study sample

We identified patients who received a new diagnosis of DM (ICD-9-CM 250) between 1 January 2000 and 31 December 2002 and included them in the DM cohort. The first outpatient or inpatient visit for the DM diagnosis during the study period was considered as the index date. Patients without DM were randomly selected at a 1:4 ratio and were included in the non-DM cohort.

To investigate the effect of incident hypertension (ICD-9-CM 401–405), incident hyperlipidemia (ICD-9-CM 272), or both, on dementia risk, we divided the two cohorts into the following four groups: patients with both hypertension and hyperlipidemia; those with only hypertension; those with only hyperlipidemia; and those without hypertension or hyperlipidemia. In both cohorts, hypertension and hyperlipidemia diagnoses were observed only following the index date during the follow-up period. To eliminate the influence of immortal time bias, the index date was set as the first date when patients received a diagnosis of hypertension or hyperlipidemia when examining the relationship of hypertension, hyperlipidemia, or both, with dementia risk.

The study endpoint was the subsequent development of dementia (ICD-9-CM 290.0–290.4, 294.1, 331.0, and 331.1–331.2) [28] from the index date until the end of 2009. To ensure validity, we excluded patients who did not have at least three ambulatory visits or one inpatient visit for the dementia diagnosis.

Moreover, we excluded patients who were younger than 20 years or older than 99 years, those with missing information regarding sex and insurance amount, and those with a history of DM and dementia. In addition, to investigate the effect of incident hypertension or hyperlipidemia on dementia after DM diagnosis, patients who had received a diagnosis of hypertension and hyperlipidemia before the index date were excluded. Moreover, to reduce the misclassification of patients who had received a diagnosis of DM, hypertension, or hyperlipidemia, we excluded those who did not meet the following criteria: (1) at least three ambulatory visits; or (2) at least one inpatient visit; or (3) at least one outpatient visit and at least one prescription for antidiabetic (ATC A10), antihypertensive (ATC C02–C03 and C07–C09), or antihyperlipidemic drugs (ATC C10).

**Fig. 1:**
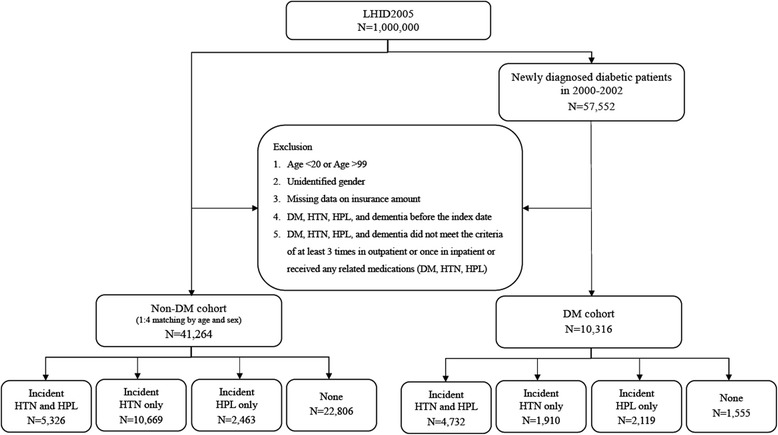
Flowchart of patient selection. *DM* diabetes mellitus, *HPL* hyperlipidemia, *HTN* hypertension, *LHID2005* Longitudinal Health Insurance Database 2005

Ultimately, 10,316 patients were enrolled in the DM cohort. The same exclusion criteria were applied to the non-DM cohort, and ultimately 41,264 patients without DM matched by age (±1 year) and sex at a 1:4 ratio with the DM cohort were enrolled in the non-DM cohort. A total of 51,580 patients were included for the final analysis. A flowchart of patient selection is presented in Fig. 1.

### Confounders

The confounders in this study were age, sex, insurance amount, and associated comorbidities. Baseline characteristics for age, sex, and insurance amount were obtained from the claims records at the index date. The presence of the following comorbidities was determined using diagnosis codes within 1 year before the index date: coronary heart disease (CHD; ICD-9-CM 410–414), stroke (ICD-9-CM 430–438), kidney disease (ICD-9-CM 580–589), atrial fibrillation (ICD-9-CM 427.3), depression (ICD-9-CM 296.2–296.3, 300.4, and 311), anxiety (ICD-9-CM 300.0, 300.2–300.3, 308.3, and 309.81 ), heart failure (ICD-9-CM 428), alcoholism (ICD-9-CM 303 and 305.0), chronic obstructive pulmonary disease (COPD; ICD-9-CM 490–496), and obesity (ICD-9-CM 278, 278.0, 278.00, and 278.01). The variables of insurance amount and COPD were used as proxies for socioeconomic status and smoking status, respectively [29]. In addition, these covariates were adjusted in the statistical models.

### Statistical analysis

For the statistical analysis, SAS Version 9.4 for Windows (SAS Institute Inc., Cary, NC, USA) was used. Continuous variables are expressed as the mean and standard deviation, and dichotomous variables are expressed as the frequency and percentage. The Mann–Whitney *U* test and chi-square test were performed to investigate the distribution of the sample characteristics and comorbidities among the various groups. The dementia-free survival rate was estimated using the Kaplan–Meier method with the examination of the log-rank test. First, dementia risk was evaluated between the DM and non-DM cohorts through Cox proportional hazards regression models with hazard ratios (HRs) and 95% confidence intervals (CIs). Moreover, multivariate Cox proportional hazards regression models were used to examine the effect of DM on dementia after adjustment for potential confounders. In addition, in both the cohorts, we examined the effect of incident hypertension or hyperlipidemia on dementia risk. Moreover, the proportions of antihypertensive or antihyperlipidemic drug prescriptions were compared between the DM and non-DM cohorts. Furthermore, we performed a sensitivity analysis by using Cox proportional hazards regression to assess the robustness of our study results by 1) adjusting confounders without heart-related diseases and stroke, and 2) analyzing patients aged between 40 and 80 years. A *p* value of less than 0.05 was considered significant.

## Results

In total, 51,580 patients aged between 20 and 99 years were enrolled. We observed 333 dementia events during 89,100.55 person-years in the DM cohort and 981 dementia events during 395,250.15 person-years in the non-DM cohort. The incidence rate of dementia was 37.37 and 24.82 per 10,000 person-years in the DM and non-DM cohorts, respectively. In the DM cohort, the percentages of type I and type II DM were 1.2% and 98.8%, respectively.

**Fig. 2:**
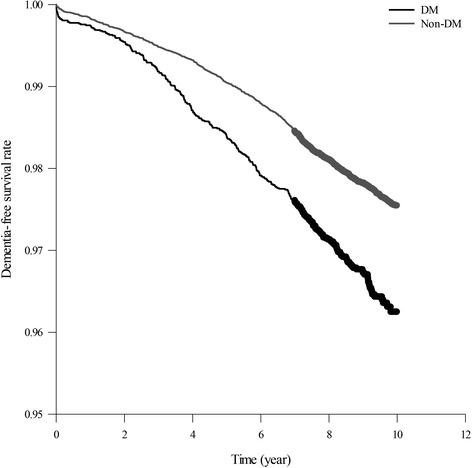
Dementia-free survival rate in patients with and without diabetes mellitus (*DM*), as estimated using the Kaplan–Meier method (log-rank test; *p* < 0.001)

The proportion of comorbidities was significantly higher in the DM cohort than in the non-DM cohort (Table 1). The Kaplan–Meier analysis revealed that the dementia-free survival rate was significantly lower in the DM cohort than in the non-DM cohort (*p* < 0.001, log-rank test; Fig. 2). The Cox proportional hazards regression model revealed that the DM cohort had a significantly higher dementia risk than the non-DM cohort (crude HR = 1.54, 95% CI = 1.36–1.74, *p* < 0.001). After adjustment for confounders, the dementia risk remained significantly higher in the DM cohort than in the non-DM cohort (adjusted HR = 1.47, 95% CI = 1.30–1.67, *p* < 0.001; Table 2).

**Table 1:** Distribution of sample characteristics according to diabetes mellitus

	Diabetes mellitus		*p* value^a^
	Yes (*n* = 10316)	No (*n* = 41264)	
Age (years), mean (SD)	53.00 (12.59)	53.00 (12.59)	1.000
Sex, *n* (%)			1.000
Male	5472 (53)	21888 (53)	
Female	4844 (47)	19376 (47)	
Coronary heart disease, *n* (%)			<0.001
Yes	592 (5.7)	819 (2)	
No	9724 (94.3)	40445 (98)	
Stroke, *n* (%)			<0.001
Yes	356 (3.5)	519 (1.3)	
No	9960 (96.5)	40745 (98.7)	
Kidney disease, *n* (%)			<0.001
Yes	444 (4.3)	455 (1.1)	
No	9872 (95.7)	40809 (98.9)	
Atrial fibrillation, *n* (%)			<0.001
Yes	39 (0.4)	52 (0.1)	
No	10277 (99.6)	41212 (99.9)	
Depression, *n* (%)			<0.001
Yes	181 (1.8)	361 (0.9)	
No	10135 (98.2)	40903 (99.1)	
Anxiety, *n* (%)			<0.001
Yes	411 (4)	649 (1.6)	
No	9905 (96)	40615 (98.4)	
Heart failure, *n* (%)			<0.001
Yes	134 (1.3)	132 (0.3)	
No	10182 (98.7)	41132 (99.7)	
Alcoholism, *n* (%)			<0.001
Yes	40 (0.4)	49 (0.1)	
No	10276 (99.6)	41215 (99.9)	
COPD, *n* (%)			<0.001
Yes	972 (9.4)	2357 (5.7)	
No	9344 (90.6)	38907 (94.3)	
Obesity, *n* (%)			<0.001
Yes	79 (0.8)	17 (0)	
No	10237 (99.2)	41247 (100)	
Insurance amount, *n* (%)			<0.001
<20000 NTD	7489 (72.6)	28545 (69.2)	
20000–40000 NTD	1602 (15.5)	6949 (16.8)	
≧40000 NTD	1225 (11.9)	5770 (14)	

*COPD* chronic obstructive pulmonary disease, *NTD* New Taiwan Dollar, *SD* standard deviation

^a^Tested using the Mann–Whitney *U* test and the chi-square test

**Table 2:** Incident hypertension and incident hyperlipidemia in prediction of dementia

		Event	PYs	Crude model			Adjusted model^b^		
				HR	95% CI	*p* value^a^	HR	95% CI	*p* value^a^
DM cohort		333	89,101	1.54	1.36–1.74	<0.001	1.47	1.30–1.67	<0.001
	Both HTN and HPL	126	24,637	1.10	0.81–1.51	0.539	0.90	0.66–1.24	0.529
	HTN only	124	11,533	2.35	1.72–3.22	<0.001	1.17	0.85–1.60	0.341
	HPL only	25	13,785	0.40	0.25–0.64	<0.001	0.72	0.45–1.17	0.189
	No HTN and HPL	58	12,932	1.00			1.00		
Non-DM cohort		981	395,250	1.00			1.00		
	Both HTN and HPL	130	24,367	2.79	2.28–3.41	<0.001	1.33	1.09–1.63	0.006
	HTN only	418	59,711	3.70	3.21–4.25	<0.001	1.22	1.05–1.40	0.008
	HPL only	31	12,468	1.31	0.90–1.89	0.154	1.28	0.89–1.85	0.187
	No HTN and HPL	402	217,378	1.00			1.00		

*CI* confidence interval, *COPD* chronic obstructive pulmonary disease, *DM* diabetes mellitus, *HTN* hypertension, *HPL* hyperlipidemia, *HR* hazard ratio, *PYs* person-years

^a^Tested using Cox proportional hazards regression

^b^Adjusted for age, sex, coronary heart disease, stroke, kidney disease, atrial fibrillation, depression, anxiety, heart failure, alcoholism, chronic obstructive pulmonary disease, obesity, and insurance amount

The distributions of age (*p* < 0.001), sex (*p* = 0.002), insurance amount (*p* < 0.001), and comorbidities of CHD (*p* < 0.001), stroke (*p* < 0.001), atrial fibrillation (*p* = 0.011), depression (*p* = 0.001), heart failure (*p* < 0.001), and COPD (*p* < 0.001) differed significantly among the four groups according to status of hypertension or hyperlipidemia in the DM cohort (data not shown). In the unadjusted model, the dementia risk in patients with both hypertension and hyperlipidemia did not increase significantly compared with that in patients without hypertension and hyperlipidemia (*p* = 0.539); however, a significantly higher risk was observed in patients with only hypertension (crude HR = 2.35, 95% CI = 1.72–3.22, *p* < 0.001), whereas there was a significantly lower risk in patients with only hyperlipidemia (crude HR = 0.40, 95% CI = 0.25–0.64, *p* < 0.001). Furthermore, after adjustment for age, sex, insurance amount, and comorbidities, the dementia risk in patients with both hypertension and hyperlipidemia did not increase significantly compared to that in patients with neither hypertension nor hyperlipidemia (*p* = 0.529). However, the nonsignificant results were observed in patients with only hypertension (*p* = 0.341) or hyperlipidemia (*p* = 0.189; Table 2), while it maintained the same direction as reported in the unadjusted model.

The results differed between the DM and non-DM cohorts. All sample characteristics differed significantly among the four groups in the non-DM cohort (all *p* < 0.05), except for alcoholism (*p* = 0.854) and obesity (*p* = 0.084; data not shown). As presented in Table 2, patients with both hypertension and hyperlipidemia exhibited a significantly higher dementia risk than those with neither hypertension nor hyperlipidemia (crude HR = 2.79, 95% CI = 2.28–3.41, *p* < 0.001). The dementia risk was also significantly higher in patients with only hypertension (crude HR = 3.70, 95% CI = 3.21–4.25, *p* < 0.001). However, patients with only hyperlipidemia did not exhibit a significantly higher dementia risk (crude HR = 1.31, 95% CI = 0.90–1.89, *p* = 0.154). The results did not change markedly after adjustment for confounders.

The proportions of antihypertensive or antihyperlipidemic treatment were examined between the DM and non-DM cohorts in patients with hypertension, hyperlipidemia, or both. No significant difference was observed in the percentage of combined prescriptions for treating hypertension and hyperlipidemia between the DM and non-DM cohort in patients with both hypertension and hyperlipidemia (99.13% and 99.62%, respectively; *p* = 0.805). In addition, the proportion of medications taken for treating hypertension in diabetic and nondiabetic patients in the hypertension-only group did not reach statistical significance (97.43% and 98.26%, respectively; *p* = 0.738). Similar results were obtained for the proportion of antihyperlipidemic drug use in patients with only hyperlipidemia between the diabetic and nondiabetic groups (69.89% and 70.32%, respectively; *p* = 0.863).

The sensitivity analysis results were comparable with the total sample, not only in the adjusted model that excluded the confounders of heart-related diseases and stroke but also in patients aged from 40 to 80 years. As shown in Table 3, the adjusted HR for dementia risk was nonsignificant in patients with hypertension, hyperlipidemia, or both compared with individuals with neither hypertension nor hyperlipidemia in patients with DM. In the non-DM cohort, an increased dementia risk was observed in patients with both hypertension and hyperlipidemia (*p* < 0.05) and hypertension only (*p* < 0.05), but not in those with hyperlipidemia only (*p* > 0.05).

**Table 3:** Sensitivity analysis of incident hypertension and incident hyperlipidemia in prediction of dementia

		Event	PYs	Adjusted model		
				HR	95% CI	*p* value^a^
Adjusted confounders without heart related diseases and stroke						
DM cohort	Both HTN and HPL^b^	126	24,637	0.90	0.65–1.24	0.514
	HTN only^b^	124	11,533	1.16	0.85–1.59	0.359
	HPL only^b^	25	13,785	0.71	0.44–1.15	0.161
	No HTN and HPL	58	12,932	1.00		
Non-DM cohort	Both HTN and HPL^b^	130	24,367	1.35	1.10–1.66	0.004
	HTN only^b^	418	59,711	1.23	1.07–1.42	0.004
	HPL only^b^	31	12,468	1.29	0.90–1.87	0.170
	No HTN and HPL	402	217,378	1.00		
Age between 40 and 80 years (*n* = 43,320)						
DM cohort	Both HTN and HPL^c^	119	22,358	0.86	0.61–1.22	0.396
	HTN only^c^	112	10,409	1.12	0.80–1.58	0.513
	HPL only^c^	25	10,657	0.75	0.46–1.23	0.255
	No HTN and HPL	48	8709	1.00		
Non-DM cohort	Both HTN and HPL^c^	127	23,444	1.33	1.07–1.64	0.009
	HTN only^c^	356	55,729	1.23	1.06–1.44	0.008
	HPL only^c^	28	11,257	1.19	0.81–1.75	0.380
	No HTN and HPL	354	166,275	1.00		

*CI* confidence interval, *COPD* chronic obstructive pulmonary disease, *DM* diabetes mellitus, *HTN* hypertension, *HPL* hyperlipidemia, *HR* hazard ratio, *PYs* person-years

^a^Tested using Cox proportional hazards regression

^b^Adjusted for age, sex, kidney disease, depression, anxiety, alcoholism, chronic obstructive pulmonary disease, obesity, and insurance amount

^c^Adjusted for age, sex, coronary heart disease, stroke, kidney disease, atrial fibrillation, depression, anxiety, heart failure, alcoholism, chronic obstructive pulmonary disease, obesity, and insurance amount

## Discussion

In this population-based retrospective cohort study, patients with incident DM exhibited a higher dementia risk during the average follow-up of 9.39 years. Although incident hypertension and incident hyperlipidemia increased the dementia risk in the non-DM cohort, the development of hypertension, hyperlipidemia, or both, following an incident diabetes diagnosis is secondary to diabetes onset and likely mediated through insulin resistance associated with diabetes, which does not further accentuate dementia risk. Moreover, we observed that, among the cardiovascular risk factors, DM itself (i.e., the systemic influence of hyperglycemia) might be the main driver of increased dementia risk.

Several studies have investigated the effect of DM on dementia in various populations worldwide [10, 12, 18, 30–32]. Our results demonstrated an association between DM and dementia risk. This finding was consistent with those of previous studies. The data source and research design used in a 2015 Taiwanese study by Kuo et al. were similar to those used in our study, except for examining the sequence of the occurrence of comorbidities [18]. However, their results were not comparable to our results because they did not adequately investigate the relationship among DM, hypertension, and hyperlipidemia. First, although they divided the diabetic cohort into insulin and noninsulin users, they did not use antidiabetic medications to define the DM cohort. By contrast, we defined strict criteria for including patients in the DM cohort, which included patients who received a DM diagnosis at least three times during outpatient visits or at least once during inpatient visits or had at least one outpatient visit and at least a prescription for antidiabetic drugs. Second, they identified comorbidities only at the baseline. Therefore, the sequence of the occurrence of comorbidities such as DM, hypertension, and hyperlipidemia remained unclear. Third, they analyzed the confounding effects of only specific variables, such as age and sex, and did not consider the effect of other comorbidities, which affected the estimation of adjusted hazard ratios. Finally, they did not evaluate the effect of hypertension or hyperlipidemia on dementia following the DM diagnosis. By contrast, we simultaneously investigated the effect of hypertension and hyperlipidemia to examine the pathogenesis underlying the dementia risk in patients with and without DM.

The exact pathogenic mechanism underlying dementia is complex and remains unclear. Our results demonstrated that the dementia risk in the DM cohort did not increase or decrease significantly in patients with hypertension, hyperlipidemia, or both. Recent studies have reported that insulin resistance might be a key factor for increasing cognitive impairment risk [10, 33]. Insulin resistance in the brain impairs insulin signaling, which can influence the regulation of food intake, body weight, reproduction, and learning and memory [34]. Disrupted insulin signaling has been associated with the development of Alzheimer’s disease [35]. It can increase senile plaques along with the deposition of amyloid beta protein and neurofibrillary tangles because of tau phosphorylation [36]. These mechanisms might accelerate the pathology of Alzheimer’s disease [33]. Insulin resistance is associated with a higher risk of type 2 DM and increases the risk of dyslipidemia and hypertension [19, 37]. In addition, insulin resistance might be the key etiological link to type 2 DM [38]. The pathogenesis underlying type 2 DM is characterized by insulin resistance and impaired insulin secretion [39]. Moreover, previous data have suggested that the development of hypertension and hyperlipidemia in patients with DM were induced by insulin resistance [22, 23]. As mentioned previously, insulin resistance is impaired by sustained hyperglycemia [7]. Therefore, we considered DM to be the main driver of the underlying dementia risk through hyperglycemia (which induces insulin resistance) rather than hypertension or hyperlipidemia.

Previous studies have reported more efficient prognosis among patients who receive medications for treating hypertension or hyperlipidemia [40, 41]. However, we observed that nondiabetic patients with hypertension and hyperlipidemia still exhibited a higher dementia risk than those without hypertension and hyperlipidemia after adjustment for potential confounders, although most individuals received related prescriptions. In the present study, the results reveal that the percentage of antihypertensive or antihyperlipidemic drugs used in the DM cohort was not significantly higher in patients with hypertension, hyperlipidemia, or both, compared with those in the non-DM cohort. Although the dementia risk varied between patients with and without DM, the medication proportions of the two cohorts were similar. Thus, this finding should not be influenced whether prescriptions are given to treat hypertension or hyperlipidemia. Otherwise, despite the high proportion of patients receiving drug prescriptions, the NHIRD does not provide detailed information regarding the medication taken. Notably, the substantial concordance for medication use between claims data and patient self-reports has been validated [42].

We observed an inverse association - although not always statistically significant - between hyperlipidemia and risk of dementia in the DM cohort which was not replicated in the non-DM one. It could be speculated that hyperlipidemia secondary to DM may exert a specific biological effect on the risk of dementia. A significantly lower dementia risk was observed in patients with diabetes and only hyperlipidemia in the crude model, whereas no significant effect was observed after adjustment for related confounders. This effect still maintained the same direction even if the significances were different before and after adjustment, which was in the absence of a significant interaction effect with all confounders. Furthermore, the lower crude HR of dementia in hyperlipidemia-only patients who received statins for at least 60 days was significant at 0.20 (*p* < 0.001) compared to those with neither hypertension nor hyperlipidemia in the DM cohort, but not in those who received statins for less than 60 days (HR = 0.56, *p* = 0.255) and in patients without statin and other antihyperlipidemic therapy (HR = 0.53, *p* = 0.057). After adjustment, only hyperlipidemia patients taking statins for at least 60 days were associated with reduced dementia risk (HR = 0.41, *p* = 0.042), while other groups showed an insignificant inverse association (HR = 1.19 and 1.02, respectively; all *p* > 0.05). In addition, a mediating effect of statins between hyperlipidemia and dementia risk was found (*p* < 0.05) in diabetic patients. A previous study reported that statin use might decrease the risk of Alzheimer’s disease [43], which might support the data interpretation. Further studies are needed to evaluate the role of hyperlipidemia and related treatments with risk of dementia in diabetic patients.

Our study has several limitations that should be addressed. First, information regarding several risk factors for dementia could not be obtained from the claims data, including information regarding cholesterol levels, family history, *APOE*, body mass index, and physical activity. In addition, the confounders extracted from medical records might be underestimated, in which the proportion of obesity was lower. This might lead to a residual effect when the association of exposures with outcomes is considered. Second, all disease information retrieved in this study was based on ICD-9-CM codes. Moreover, because the LHID2005 contains the claims data of beneficiaries enrolled in the NHI program, potential ascertainment bias and misclassification bias may be present. However, we used a strict criterion to identify the exposure and outcome to reduce the uncertainty of diagnosis. In addition, the NHI program covers approximately 99% of the residents of Taiwan and has good generalizability; therefore, the resulting misclassification bias is likely to be nondifferential. Third, surveillance bias might occur in patients with and without DM when detecting incident hypertension or hyperlipidemia. However, the percentages of antihypertensive or antihyperlipidemic prescriptions in each group were comparable between the two cohorts. Finally, mild cognitive impairment and dementia severity cannot be identified from the NHIRD. We included all dementia types; therefore, the results of different dementia severities could not be determined. However, the most common cause of dementia in Taiwan is Alzheimer’s disease, the diagnosis of which is relatively homogeneous [44]. In addition, patients with dementia before the index date were excluded in order to examine the causality.

## Conclusions

The dementia risk was higher in the DM cohort than in the non-DM cohort during the average follow-up of 9.39 years. DM itself (i.e., the systemic influence of hyperglycemia) might be the main driver of increased dementia risk. The development of hypertension, hyperlipidemia, or both, following an incident diabetes diagnosis is secondary to diabetes onset and likely mediated through the insulin resistance associated with diabetes, which does not further accentuate dementia risk. However, hypertension and hyperlipidemia increased the dementia risk in the non-DM cohort. Future studies should examine the pathogenesis underlying dementia. The prevention of these comorbidities during diabetes remains clinically crucial and might reduce the dementia risk.

## Abbreviations

ATC: Anatomical Therapeutic Chemical
CHD: Coronary heart disease
CI: Confidence interval
COPD: Chronic obstructive pulmonary disease
DM: Diabetes mellitus
HR: Hazard ratio
ICD-9-CM: International Classification Of Diseases, Ninth Revision, Clinical Modification
LHID2005: Longitudinal Health Insurance Database 2005
NHI: National Health Insurance
NHIRD: National Health Insurance Research Database
NHRI: National Health Research Institutes
